# Clinical Utility of Functional Precision Medicine in the Management of Recurrent/Relapsed Childhood Rhabdomyosarcoma

**DOI:** 10.1200/PO.20.00438

**Published:** 2021-10-27

**Authors:** Arlet M. Acanda De La Rocha, Maggie Fader, Ebony R. Coats, Paula S. Espinal, Vanessa Berrios, Cima Saghira, Ileana Sotto, Rojesh Shakya, Michelin Janvier, Ziad Khatib, Haneen Abdella, Mathew Bittle, Cristina M. Andrade-Feraud, Tomás R. Guilarte, Jennifer McCafferty-Fernandez, Daria Salyakina, Diana J. Azzam

**Affiliations:** ^1^ Department of Environmental Health Sciences, Robert Stempel College of Public Health & Social Work, Florida International University, Miami, FL; ^2^ Personalized Medicine Initiative, Nicklaus Children's Hospital, Miami, FL; ^3^ Pediatric Oncology and Hematology, Nicklaus Children's Hospital, Miami, FL; ^4^ Miller School of Medicine, University of Miami, Miami, FL

## Introduction

**FIG 1.**
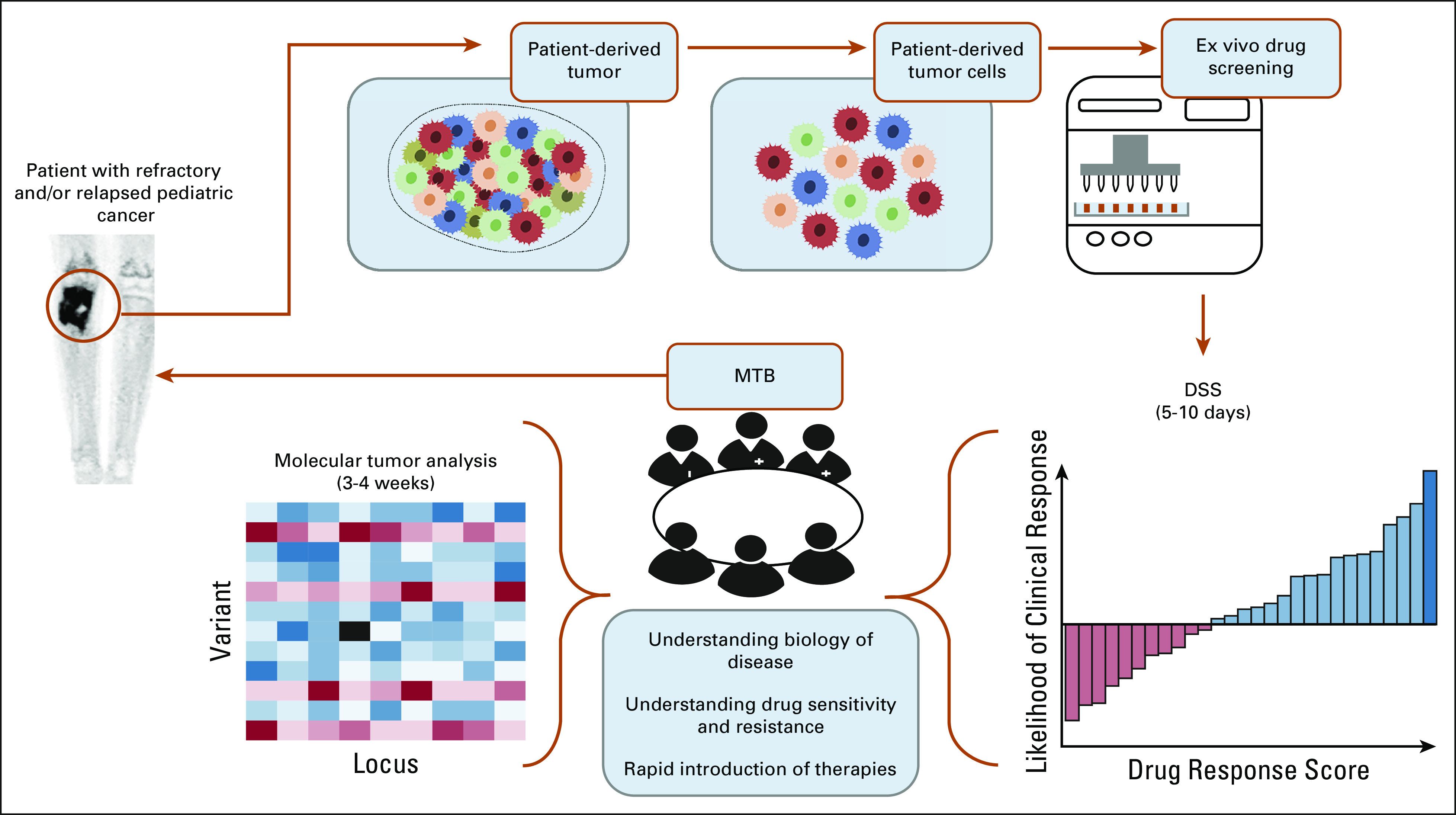
Functional precision medicine in the management of recurrent childhood cancers. Illustration of our functional precision medicine program depicting the workflow, beginning with the sample collection, and processing, continuing with the establishment of patient-derived tumor cells that are subjected to single drug testing. In parallel, DNA is sent for targeted gene sequencing (UCSF500). The DSS are reported back to the MTB within 5-10 days, whereas the sequencing data take up to 3 weeks. The MTB reviews the data, likely off-label availability for candidate drugs, prior experience with candidate drugs, and treatment history of each patient. A final list of therapeutic options (ranked in order of preference together with suggested doses and schedules) is issued, and patients are treated with the best recommendations whenever possible. DSS, drug sensitivity score; MTB, Molecular Tumor Board.

Treatments for rhabdomyosarcoma (RMS) including sclerosing and spindle cell rhabdomyosarcoma (SRMS) remains challenging. Aggressive surgical resection and radiation therapy are generally recommended; however, there is no consensus on second- or third-line salvage chemotherapy.^1^ Therefore, developing novel therapeutic options for children with all types of RMS suffering relapse under standard protocols is vital. Here, we present results using our functional precision medicine platform in a heavily refractory pediatric SRMS patient who had exhausted all standard options. Our functional precision medicine platform integrates genomic profiling and ex vivo drug sensitivity testing (DST) to determine optimal individualized therapy for each patient (Fig 1).^2^

## Case Report

**FIG 2.**
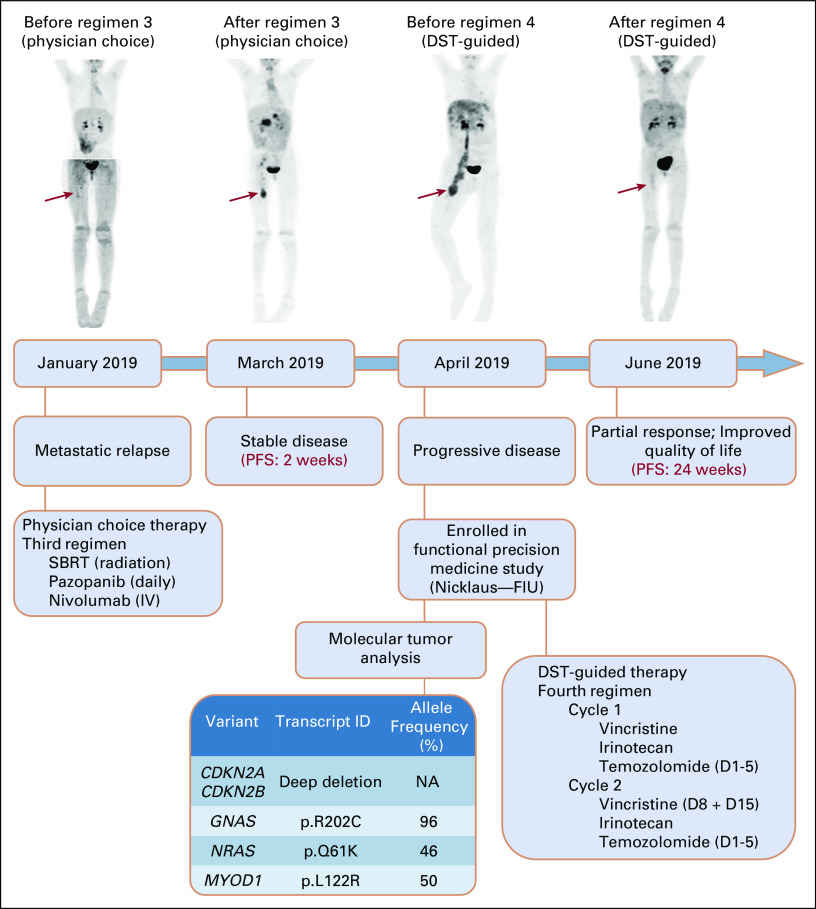
Clinical course and response images of relapsed SRMS patient. Upper panel shows the timeline of treatments after relapse and progression in the patient. PET scans were obtained at relapse (January 2019) and after regimen 3 (March 2019), as well as before (April 2019) and after (June 2019) fourth-line DST-guided therapy. Before DST-guided therapy, there was progressive disease with PET demonstrating a large, FDG-avid right mass in the lower extremity with a right pelvic mass, right thigh lymph node, and right lung nodule (April 2019). PET scan obtained after 8 weeks of DST-guided therapy in June 2019 (vincristine in combination with irinotecan and temozolomide) demonstrated significantly decreased size of ill-defined soft tissue mass seen encasing the femoral neurovascular bundle (residual lower-level FDG uptake in the right thigh). In addition, interval improvement was observed in disease burden with residual masses throughout liver parenchyma, pancreatic head, and body as well as stable right middle lobe pulmonary nodule. Bottom panel shows results from our functional precision medicine platform (molecular tumor analysis and drug sensitivity testing) on the recurrent tumor. D, day; DST, drug sensitivity testing; FDG, fluorodeoxyglucose; FIU, Florida International University; IV, intravenous; NA, not available; PET, positron emission tomography; PFS, progression-free survival; SBRT, stereotactic body radiation therapy; SRMS, sclerosing and spindle cell rhabdomyosarcoma.

**FIG 3.**
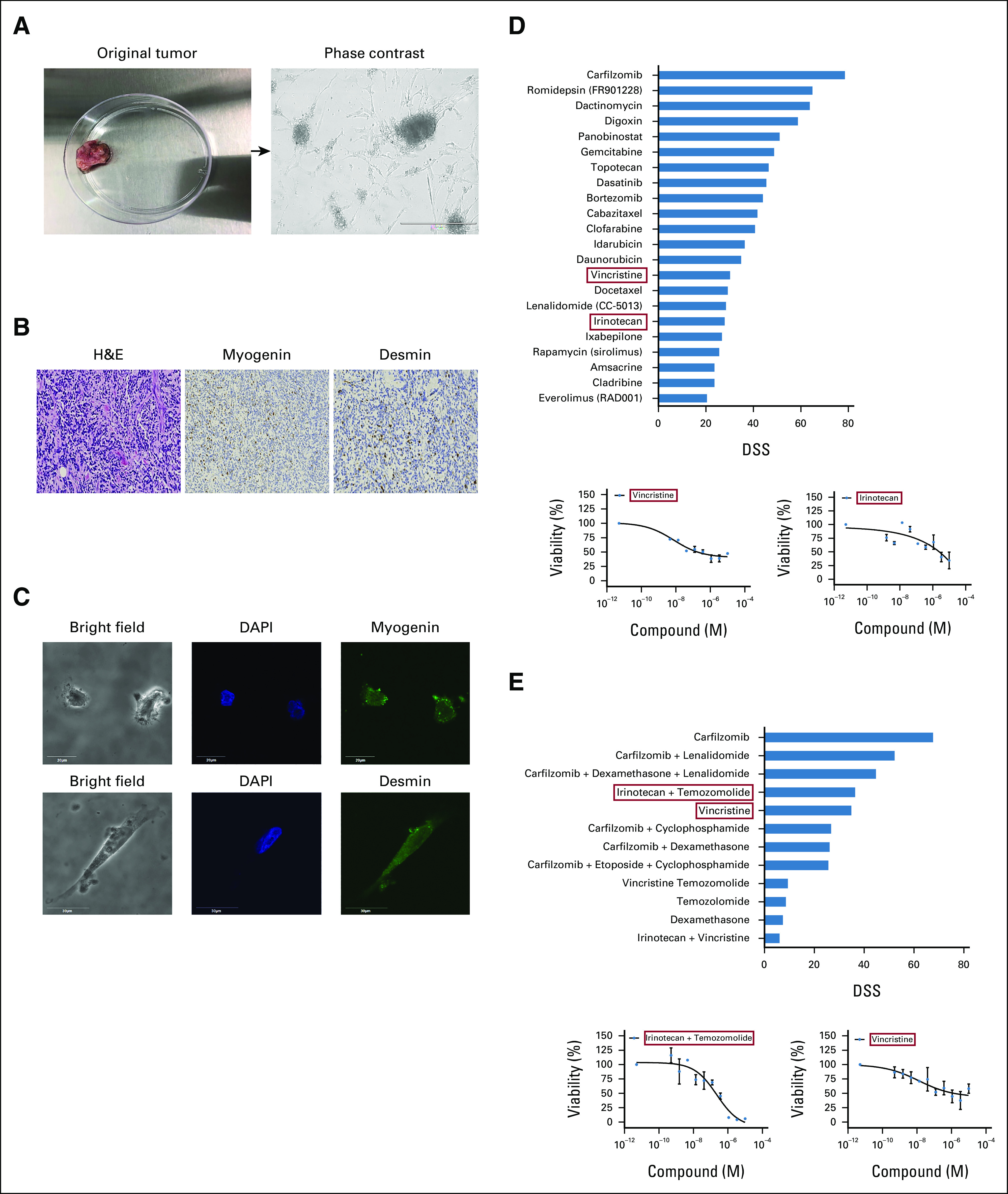
Ex vivo drug sensitivity results identify top effective drugs for treatment. (A) Representative image of original RMS tumor (left) and patient-derived tumor cells (right) as visualized under light microscope. Scale bars represent 400 µm, magnification 10×. (B) Immunohistochemical staining from the tumor tissue, H&E (left), myogenin (middle), and desmin (right) magnification 100×. (C) Immunofluorescence images show patient-derived cells express RMS markers, myogenin and desmin, scale bar is 20 µm, magnification 144×. (D) Top panel shows graph plot that highlights the most effective and potent drugs on the DST performed. Drugs selected for therapy are highlighted in red boxes. The bottom panel shows dose-response curves of vincristine and irinotecan. (E) The top panel shows most effective combination drugs including irinotecan and temozolomide in combination, as well as vincristine as a single agent (red boxes). The bottom panel shows the dose-response curves of the selected drugs highlighted in red boxes. The patient treatment regimen was modified based on DST results. DAPI, 4’,6-diamidino-2-phenylindole; DSS, drug sensitivity score; DST, drug sensitivity testing; H&E, hematoxylin and eosin; RMS, rhabdomyosarcoma.

A 7-year-old girl initially presented in August 2016 with SRMS in her lower right extremity at Nicklaus Children's Hospital in Miami, FL. She underwent surgery, followed by 6 weeks of external beam radiation therapy and vincristine, irinotecan, doxorubicin, cyclophosphamide, ifosfamide, and etoposide for 52 weeks. Six months later, positron emission tomography (PET) imaging revealed a 6-cm lower right quadrant mass, a 1-cm right femur lymph node that was avid, and a single 3-mm pulmonary nodule. The patient commenced second-line treatment comprising temsirolimus, vinorelbine, and cyclophosphamide (progression-free survival [PFS]: 30 weeks). In January 2019, radiologic assessment of disease revealed resolution of the pelvic mass but marked a new hepatic lesion, indicative of metastasis (see Fig 2 showing the clinical course of disease relapses and progression). Third-line treatment was started, consisting of oral pazopanib daily plus nivolumab intravenously combined with external radiation therapy. Nevertheless, disease progression continued to refractory disease 2 weeks following regimen completion (PFS: 2 weeks). The patient had severe swelling and lymphedema of her right lower extremity, abdominal distention, pain, and coagulopathy. By March 2019, she presented with recurrent metastatic SRMS (stage III, group III high risk). PET, in combination with computed tomography (CT) images, revealed a right thigh mass encasing the femoral artery and neurovascular bundle including multiple metastatic nodes in the right pelvis, right thigh lymph node, liver, and pancreas. Given her rapid disease progression, alternative therapies were sought. In April 2019, the patient was enrolled in our ongoing clinical trial (Institutional Review Board approval number 1186919, ClinicalTrials.gov identifier: NCT03860376, patient provided informed consent, including consent to publish). Our trial involves high-throughput DST of 103 clinically approved drugs combined with targeted mutation profiling to guide clinical decision in relapsed and/or refractory pediatric cancer patients. The DST panel encompassed 40 formulary drugs used at Nicklaus Children's Hospital, 47 nonformulary US Food and Drug Administration–approved cancer treatment drugs, and drugs from phase III or IV clinical trials (Table 1). Biopsy was performed on the right proximal anterior thigh, from which excised tumor was obtained to isolate cells (Fig 3A) within 24 hours, as previously described.^3^ Histologic examination showed spindle cells with fusiform nuclei, arranged in fascicles in a background of sclerosing stroma with islands of dense hyalinized collagen along with strong nuclear myogenin and cytoplasmic desmin positivity, typical of RMS (Fig 3B).^4-6^ We also confirmed that patient-derived cells used for DST expressed myogenin and desmin, similar to the originating tumor (Fig 3C). Cells were cultured in 384-well plates, and then interrogated for DST panel sensitivity by measuring the number of metabolically active tumor cells 72 hours after drug exposure. A ranked list of the most potentially therapeutic drugs was generated using dose-response curves, as previously described,^7,8^ and communicated to the Molecular Tumor Board within 7 days postsurgery (Fig 3D).

**TABLE 1.**
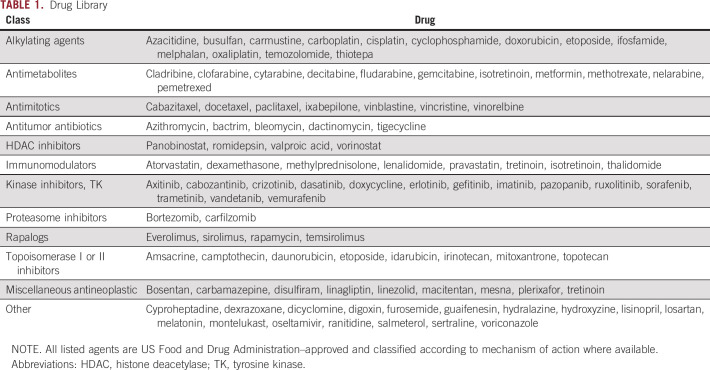
Drug Library

Blood and tumor samples were also collected for mutational profile analysis using the UCSF500 Cancer Gene Panel Test (University of California San Francisco). As profiling took up to 3 weeks, the Molecular Tumor Board first analyzed DST results to choose effective, available drug options (Fig 3D) to begin patient treatment. Interestingly, the microtubule destabilizer vincristine and topoisomerase inhibitor irinotecan showed up as two effective drugs. Both drugs had lower half-maximal effective concentrations (EC50) than the reported maximum concentration (C_max_) in the plasma of the patient, hence they were clinically achievable. Given that a combination of vincristine, irinotecan, and temozolomide (VIT) is often used to treat pediatric relapsed RMS,^9^ the patient was immediately treated with one cycle of VIT from days 1 to 5. Importantly, all drugs used in the patient's previous regimens were ineffective (drug sensitivity scores, ≤ 0) by DST, except vincristine and irinotecan, which both presented sustained activity on the tumor, supporting DST-guided fourth-line treatment (Data Supplement). Additionally, to identify optimal combination therapies, a secondary combination DST was performed using drugs with the best individual scores. Drug sensitivity score for vincristine alone were higher than in combination with temozolomide or irinotecan (Fig 3E). As such, treatment was modified: the patient received three cycles of vincristine alone (1.5 mg/m^2^, bolus) on days 8 and 15, followed by a combination of irinotecan (50 mg/m^2^, intravenous) and temozolomide (125 mg/m^2^, oral) from days 1 to 5.

Results from patient mutational profiling showed a deep deletion in *CDKN2A* and *CDKN2B*, a heterozygous L122R mutation in *MYOD1* exon1 hotspot*,* and new single-point pathogenic mutations in *GNAS* and *NRAS* (Fig 2). The patient was *PAX3*-*FOXO1* and *PAX7*-*FOXO1* fusion-negative, which are typical genetic hallmarks of alveolar RMS. The hotspot *MYOD1* mutation is a recurrent finding in pediatric SRMS, especially in tumors from lower-extremity sites,^10^ and is associated with a highly aggressive clinical course despite multimodality therapies.^11-14^ *MYOD1* gene regulates muscle cell determination and differentiation, by inducing cell cycle arrest.^15^ *GNAS* mutations, interestingly, have been associated with numerous neoplasms, including Ewing sarcoma,^16^ but have not yet been linked to SRMS. A recent report in RMS has shown that *NRAS* mutation is one of the most frequently observed genetic alterations in fusion-negative tumors. Additionally, a mutation of a Ras pathway gene is found in 56% of all fusion-negative tumors (N = 515 samples).^17^ The selection of drugs targeting cell division was appropriate, as the patient's molecular profile involved alterations to myogenic differentiation, cell cycle progression, cell division, and Ras signaling. The heterozygous *MYOD1* mutation was also found on the original tumor, confirming that the same genetic abnormality was persistent and allele frequency did not change during treatment course. In the first follow-up visit, 8 weeks following DST-guided treatment, PET imaging showed a striking decrease in the ill-defined soft tissue mass in the medial right proximal thigh and decreased nodular density intermixed with linear densities in the adjacent subcutaneous tissues of the anterior right thigh (Fig 2). These results showed a decrease in tumor burden, indicative of a partial response according to RECIST.^18^ Overall metastatic disease in the liver and pancreas also decreased. Remarkably, the patient was discharged only 10 days following DST-guided therapy commencement. Her clinical symptoms and quality of life improved dramatically. The overall burden of lung disease was stable. The patient remained clinically disease-free for 6 months (PFS: 24 weeks), compared to 2 weeks with prior regimen (Fig 2). Unfortunately, despite other DST-guided options, pulmonary nodules progressed, leading to lung collapse. The patient ultimately received no further therapy and died approximately 8 months following study enrollment.

## Discussion

Recurrent and metastatic SRMS has a poor prognosis, with survival often measured in months (median 2-4 months).^19^ This clinical case shows the significance of developing a functional precision oncology approach, using patient material for DST—with confirmation by molecular profiling—to identify the most effective drug combination for a child with recurrent metastatic SRMS. Clinically and radiographically, this patient showed a positive response to DST-guided treatment, despite previous progression with standard therapy. Time to progression (TTP; 6 months) was considerably longer than prior regimen (TTP: 2 weeks) or most standard fourth-line regimens for relapsed and/or refractory RMS (median TTP: 2-4 months, including patients with less aggressive disease).^20^

DST results were ready in less than a week for quick treatment adoption by oncologists. Although molecular profiling took 3 weeks, results showed mutations in cell cycle and cell division regulatory genes, as well as the Ras signaling pathway, and supported DST-guided treatment. Notably, genetic profile indicated a deletion in *CDKN2A* and *CDKN2B* genes along with *MYOD1* mutation, which is consistent with a recent report showing *MYOD1*-mutant tumors also frequently harbored deep deletions in *CDKN2A* in patients with fusion-negative RMS.^17^ This highlights the importance of evaluating *MYOD1* and *CDKN2A* alterations in the genetic analysis, taking into consideration that *MYOD1* L122 hotspot is not generally included on most institution panel sequencing. A negative sequencing result does not always mean that the mutation has been assayed.

VIT therapy showed a significant clinical benefit, as evidenced by decreased tumor size. VIT is sometimes used to treat pediatric relapsed RMS; however, PFS at 3 months is 23% (95% CI, 5.7 to 46.7).^9^ The treating oncologist would have not decided to use that regimen without guidance from the drug testing results especially because two of those drugs were used in upfront therapy (3 years prior). However, the clinical benefit shown on the patient corresponds to the positive activity of those drugs in the DST that was performed ex vivo.

Although several reports have shown improved patient survival using DST-guided treatment in different cancers,^21-24^ current DST platforms have a limited ability to study the immune system and tumor microenvironment. There are also pharmacologic limitations such as prodrug formulations, liver-metabolized drugs, and pharmacokinetic information obtained in adults receiving different doses than pediatric patients. Vincristine is given as a bolus, briefly reaching high concentrations in the bloodstream, whereas irinotecan and temozolomide are given over 5 days, with high concentrations each day; this highlights the difference between in vivo and ex vivo assays. The ultimate negative outcome of the patient underscores the need for DST of tumor cells from several sites, including metastatic sites, to better inform treatment.

In summary, this study demonstrates that a personalized functional precision oncology approach resulted in a longer PFS on a DST-suggested regimen than on the regimen on which the patient had just experienced progression. DST offers an assessment of additional possible treatments and combinations to identify effective therapeutic strategies for patients. Most importantly, high-throughput DST can be done in a clinically relevant timeframe, is individualized, and can be performed throughout the disease course. Combining genomics and functional data further refines precision therapy, enhancing appropriate drug combination selection—even lacking actionable biomarkers—and ultimately improving clinical outcomes for pediatric patients with relapsed and/or recurrent tumors, establishing a new treatment paradigm in pediatric cancer.

## References

[1] Ray A, Huh WW: Current state-of-the-art systemic therapy for pediatric soft tissue sarcomas. Curr Oncol Rep 14:311-319, 2012 22535507 10.1007/s11912-012-0243-y

[2] Letai A: Functional precision cancer medicine-moving beyond pure genomics. Nat Med 23:1028-1035, 2017 28886003 10.1038/nm.4389

[3] Brodin BA, Wennerberg K, Lidbrink E, et al: Drug sensitivity testing on patient-derived sarcoma cells predicts patient response to treatment and identifies c-Sarc inhibitors as active drugs for translocation sarcomas. Br J Cancer 120:435-443, 2019 30745580 10.1038/s41416-018-0359-4 PMC6462037

[4] Altmannsberger M, Weber K, Droste R, et al: Desmin is a specific marker for rhabdomyosarcomas of human and rat origin. Am J Pathol 118:85-95, 1985 3881039 PMC1887849

[5] Kodet R: Rhabdomyosarcoma in childhood. An immunohistological analysis with myoglobin, desmin and vimentin. Pathol Res Pract 185:207-213, 1989 2798221 10.1016/S0344-0338(89)80253-5

[6] Kumar S, Perlman E, Harris CA, et al: Myogenin is a specific marker for rhabdomyosarcoma: An immunohistochemical study in paraffin-embedded tissues. Mod Pathol 13:988-993, 2000 11007039 10.1038/modpathol.3880179

[7] Lohse I, Azzam DJ, Al-Ali H, et al: Ovarian cancer treatment stratification using ex vivo drug sensitivity testing. Anticancer Res 39:4023-4030, 2019 31366484 10.21873/anticanres.13558 PMC7323502

[8] Swords RT, Azzam D, Al-Ali H, et al: Ex-vivo sensitivity profiling to guide clinical decision making in acute myeloid leukemia: A pilot study. Leuk Res 64:34-41, 2018 29175379 10.1016/j.leukres.2017.11.008 PMC5756519

[9] Setty BA, Stanek JR, Mascarenhas L, et al: VIncristine, irinotecan, and temozolomide in children and adolescents with relapsed rhabdomyosarcoma. Pediatr Blood Cancer 65:e26728, 2018 10.1002/pbc.26728 PMC7497851 28748602

[10] Szuhai K, de Jong D, Leung WY, et al: Transactivating mutation of the MYOD1 gene is a frequent event in adult spindle cell rhabdomyosarcoma. J Pathol 232:300-307, 2014 24272621 10.1002/path.4307

[11] Owosho AA, Chen S, Kashikar S, et al: Clinical and molecular heterogeneity of head and neck spindle cell and sclerosing rhabdomyosarcoma. Oral Oncol 58:e6-e11, 2016 27261172 10.1016/j.oraloncology.2016.05.009 PMC5518412

[12] Agaram NP, Chen CL, Zhang L, et al: Recurrent MYOD1 mutations in pediatric and adult sclerosing and spindle cell rhabdomyosarcomas: Evidence for a common pathogenesis. Genes Chromosomes Cancer 53:779-787, 2014 24824843 10.1002/gcc.22187 PMC4108340

[13] Rekhi B, Upadhyay P, Ramteke MP, et al: MYOD1 (L122R) mutations are associated with spindle cell and sclerosing rhabdomyosarcomas with aggressive clinical outcomes. Mod Pathol 29:1532-1540, 2016 27562493 10.1038/modpathol.2016.144 PMC5133269

[14] Agaram NP, LaQuaglia MP, Alaggio R, et al: MYOD1-mutant spindle cell and sclerosing rhabdomyosarcoma: An aggressive subtype irrespective of age. A reappraisal for molecular classification and risk stratification. Mod Pathol 32:27-36, 2019 10.1038/s41379-018-0120-9 PMC6720105 30181563

[15] Tapscott SJ: The circuitry of a master switch: Myod and the regulation of skeletal muscle gene transcription. Development 132:2685-2695, 2005 15930108 10.1242/dev.01874

[16] Noh BJ, Sung JY, Kim YW, et al: Clinicopathological implications of GNAS in Ewing sarcoma. Oncol Lett 11:4077-4082, 2016 27313744 10.3892/ol.2016.4521 PMC4888216

[17] Shern JF, Selfe J, Izquierdo E, et al: Genomic classification and clinical outcome in rhabdomyosarcoma: A report from an International Consortium. J Clin Oncol 39:2859-2871, 2021 34166060 10.1200/JCO.20.03060 PMC8425837

[18] Eisenhauer EA, Therasse P, Bogaerts J, et al: New response evaluation criteria in solid tumours: Revised RECIST guideline (version 1.1). Eur J Cancer 45:228-247, 2009 19097774 10.1016/j.ejca.2008.10.026

[19] Breneman JC, Lyden E, Pappo AS, et al: Prognostic factors and clinical outcomes in children and adolescents with metastatic rhabdomyosarcoma—A report from the Intergroup Rhabdomyosarcoma Study IV. J Clin Oncol 21:78-84, 2003 12506174 10.1200/JCO.2003.06.129

[20] PDQ: Childhood Rhabdomyosarcoma Treatment (PDQ®): Health Professional Version. Bethesda, MD, PDQ Cancer Information Summaries, 2020

[21] Gazdar AF, Steinberg SM, Russell EK, et al: Correlation of in vitro drug-sensitivity testing results with response to chemotherapy and survival in extensive-stage small cell lung cancer: A prospective clinical trial. J Natl Cancer Inst 82:117-124, 1990 2152944 10.1093/jnci/82.2.117

[22] Iwadate Y, Fujimoto S, Namba H, et al: Promising survival for patients with glioblastoma multiforme treated with individualised chemotherapy based on in vitro drug sensitivity testing. Br J Cancer 89:1896-1900, 2003 14612899 10.1038/sj.bjc.6601376 PMC2394441

[23] Wu B, Zhu JS, Zhang Y, et al: Predictive value of MTT assay as an in vitro chemosensitivity testing for gastric cancer: One institution's experience. World J Gastroenterol 14:3064-3068, 2008 18494060 10.3748/wjg.14.3064 PMC2712176

[24] Snijder B, Vladimer GI, Krall N, et al: Image-based ex-vivo drug screening for patients with aggressive haematological malignancies: Interim results from a single-arm, open-label, pilot study. Lancet Haematol 4:e595-e606, 2017 29153976 10.1016/S2352-3026(17)30208-9 PMC5719985

